# Home-Based Occupational Therapy for Emotion-Related School Attendance Challenges: Two Case Studies

**DOI:** 10.7759/cureus.99782

**Published:** 2025-12-21

**Authors:** Fugen Oto, Naoko Matsuda, Kaori Ito, Atsuko Morikawa, Hiromi Fujii

**Affiliations:** 1 Rehabilitation, Iroha Visiting Nurse Rehabilitation Station, Hiroshima, JPN; 2 Occupational Therapy, Graduate School, Yamagata Prefectural University of Health Sciences, Yamagata, JPN; 3 Rehabilitation, Developmental Support Room Mifaso, Hiroshima, JPN; 4 Rehabilitation, Kanon Co. Ltd, Hiroshima, JPN

**Keywords:** adolescent mental health, emotion-related school attendance challenges (er-sac), home-based occupational therapy, school nonattendance, social reintegration

## Abstract

Emotion-related school attendance challenges (ER-SAC) refer to school attendance difficulties rooted in emotional issues. This report examined the support process and outcomes of home-based occupational therapy in two junior high school students experiencing long-term absenteeism and social withdrawal. Both students exhibited emotional difficulties, including anxiety, interpersonal tension, and impulsivity, that disrupted daily routines, impaired self-care, and led to social avoidance. Occupational therapists initiated home visits by building trust, then gradually introduced strategies to restructure daily routines, verbalize emotions, enhance self-understanding, and set interest-based goals. Both cases demonstrated improvements in interpersonal relationships, leisure activities, and coping skills. In Case 1, vocational exploration and growing introspection fostered future aspirations. In Case 2, reduced impulsivity and mistrust improved social skills, enabling desired participation in club and sports activities. Both students progressed from "blockage and isolation" to "social connection and active participation". Home-based occupational therapy appears effective for life restructuring and social reintegration in students with ER-SAC. The support model offers practical guidance for similar student cases, emphasizing the need for flexible, individualized plans considering emotional, cognitive, and social characteristics over uniform interventions.

## Introduction

In recent years, school nonattendance has emerged as a serious social issue in many countries, including Japan [1,2]. According to the Ministry of Education, Culture, Sports, Science and Technology, *Futoko* or long-term school absenteeism in Japan refers to children absent from school for 30 or more days annually due to psychological, emotional, physical, or social factors, excluding illness or economic reasons [3]. In the 2023 academic year, the number of such absentees in elementary and junior high schools surpassed 346,000, marking an 11-year consecutive rise [3]. This trend underscores an urgent need for effective support strategies for long-term absenteeism.

School nonattendance is increasingly viewed as a multifaceted educational, psychological, and social issue, prompting reexamination of its terminology. Traditional terms like "school refusal" and "school phobia" have been criticized for placing undue blame on the child and failing to reflect underlying complexities [4]. Recently, the term Emotion-Related School Attendance Challenges (ER-SAC) has been proposed as a more inclusive and respectful alternative that centers the perspectives of both students and guardians [5,6]. ER-SAC conceptualizes attendance difficulties as rooted primarily in emotional factors, encompassing behaviors previously classified as "school refusal" and promoting more person-centered language. Accordingly, this report refers to school absenteeism and similar terms as ER-SAC.

Schools identify numerous reasons for ER-SAC, including low motivation, anxiety, depression, disrupted routines, academic underperformance, and peer issues [7,8]. Studies suggest that children with anxiety symptoms or neurodevelopmental disorders face higher risks of maladjustment and ER-SAC [8,9]. In particular, students with autism spectrum disorder (ASD) and attention-deficit/hyperactivity disorder (ADHD) often struggle with school adaptation and are more likely to avoid school [10,11]. Difficulties in social communication, sensory processing, and emotional regulation heighten stress and anxiety, contributing to withdrawal. Emotional conditions such as social anxiety, generalized anxiety disorder, and past trauma are also deeply linked to ER-SAC behavior [12]. In light of this, highly individualized support with mental health considerations is essential. Occupational therapy (OT) may offer a promising approach. Nevertheless, few practical reports highlight OT’s strengths, such as client-centered interventions grounded in daily life and flexible, stepwise strategies tailored to psychological and developmental needs. Continuous home-visit OT case studies remain scarce.

Since 2015, the authors have provided home-based OT for children with ER-SAC through Japan’s Health Insurance Act system. Prior research documented that 13 Occupational Therapists Registered (OTRs) supported 108 children, with 77 resuming school attendance [13]. In a subsequent work, we proposed three broad categories for ER-SAC cases and reported one representative case from the most prevalent group [14]. The current report presents two additional cases, describing how home-based OT supported daily routine rebuilding, emotional expression, coping skill development, and future role formation. It further explores OT’s potential for supporting not only school reintegration but also medium- and long-term social participation and occupational identity development.

Based on available literature, this is the first report from Japan to examine the effectiveness of home-based OT for adolescents with ER-SAC, using the CHIME (Connectedness, Hope and optimism, Identity, Meaning in life, and Empowerment) recovery model and multidimensional outcome measures [15]. This report presents a structured and phased intervention process, from rebuilding daily routines to emotional expression and social reintegration, tailored to each student’s unique psychosocial profile. By comparing two contrasting cases, this study emphasizes the importance of individualized, flexible OT planning rather than standardized approaches, thus offering a novel and practical framework for supporting ER-SAC students in real-world clinical settings.

## Case presentation

This report presents two male students who experienced junior high school nonattendance and received home-based OT. The selection of these cases was based on a cluster analysis of the Japanese version of the Vineland Adaptive Behavior Scales, Second Edition (Vineland-II) [16], a standardized and validated instrument, conducted with students with ER-SAC who were currently receiving home-based OT. The Japanese version of the Vineland-II used for these cases was the officially authorized version published by Nihon Bunka Kagaku Sha, purchased through an authorized distributor in Japan.

The two cases presented in this report belonged to distinctly different clusters. Both received support through our affiliated visiting nurse station. The study’s purpose, methods, and confidentiality measures were explained verbally and in writing to the students and their guardians, and written consent was obtained.

The Vineland-II (Japanese version) was used to evaluate social functioning and adaptive behavior. This standardized scale measures adaptive behavior in children with developmental and emotional challenges. We focused on the "Socialization Domain" and "Maladaptive Behavior Domain." The Socialization Domain includes interpersonal relationships, leisure activities, and coping skills, making it highly relevant to this study’s goals. Scores are rated on a 3-point scale: 2 (usually does), 1 (sometimes does), and 0 (never does). Additionally, the Maladaptive Behavior Domain assessed the presence and frequency of internalizing and externalizing behaviors, as well as social deviance. It uses a reversed scoring scale: 2 (always present), 1 (sometimes present), and 0 (never present). Subdomain results are expressed as v-scores (M = 15, SD = 3).

Additionally, OTRs recorded verbal expressions, behavioral changes, and guardian feedback during visits to capture qualitative changes. Social participation, including school attendance and education options, was discussed in collaboration with guardians and school teachers.

Case study 1

A 13-year-old male child living with his parents and younger sister was diagnosed with social anxiety disorder. His physician noted that the patient "experiences fear and refusal toward school, resulting in continued social isolation. Though cognitively capable, he cannot communicate his future plans to his family. Third-party intervention is necessary.”

Home visits began in May of his second year of junior high school. The child had studied rigorously in elementary school and passed an entrance exam to a high-performing private junior high. However, after the summer of his first year, he stopped attending and did not respond to the school’s efforts. His parents sought medical help, and a physician recommended home-based OT. The child’s social anxiety traits, support process, and parental dynamics are summarized in Table 1. OTR visits focused on re-establishing a morning routine. Within a month, he began expressing academic stress, saying, “I’m trying my best, but my grades aren’t improving.” He also began discussing school and test-related difficulties with the OTR, who empathized and shared school experiences.

**Table 1:** Intervention process for the child and his parents (Case 1) OTR: Occupational Therapists Registered

Phase	Period	Key Characteristics and Challenges in Case 1		OTR Intervention Goals and Plan	OTR Intervention and Patient Response	Support for and Relationship with Family (Parents)
Phase 1	May, 2nd Year of Junior High (1st & 2nd visits)	Exhibited severe anxiety symptoms and social withdrawal; refused interpersonal interaction outside of family and OT sessions.	Goal: Make child aware of the home visits. Plan: OTR met the child and his parents during visits. After each session, parents were encouraged to talk about the OTR’s personality and conversation topics. OTR also conducted interviews with parents to identify opportunities for direct engagement.		After a few visits, the child responded cheerfully when the OTR knocked and allowed them in. Though restless, he engaged when the OTR discussed his favorite games.	OTR shared updates with parents and explained common symptoms of social anxiety and its impact on daily functioning, promoting understanding.
Phase 2	June–September, 2nd Year of Junior High	Tended to stay in his room all day with a disrupted daily rhythm. Frequently displayed compulsive hand-washing behavior.	Goal: Establish a secure relationship and adjust daily rhythm. Plan: Scheduled visits for 1 PM. Interventions included listening to his favorite game topics and academic struggles while empathizing. Emphasized that engaging with OTR was itself a form of social participation.		Initially, he was sometimes asleep during visits, but later began waiting for the OTR. He stopped refusing visits and expressed a desire for academic recognition. As sessions progressed, he smiled more and showed interest in the OT profession, asking about qualifications.	The parents, both teachers, were anxious about the child’s future and concerned about his lack of academic interest. OTR framed his symptoms as medical, encouraging parental acceptance and suggesting this was a "rest period." To support their understanding, the OTR also shared past experiences working with other students who benefited from a similar approach, which helped the parents view the “rest period” as a meaningful therapeutic phase rather than a setback.
Phase 3	October to March, 2nd Year of Junior High	Perfectionistic and proud, which led to self-blame over academic ranking, heightened study-related anxiety, and school maladjustment. Viewed his own limitations negatively, resulting in very low self-efficacy.	Goal: Promote self-esteem and social engagement. Plan: Discussed future aspirations in a study-supportive environment. Suggested cram school as a preliminary step toward reintegration.		He began showing old test papers to the OTR and expanded discussions around education. He talked about going to a festival with elementary school friends and expressed enjoyment of karaoke.	OTR conveyedthe child’s desire to resume studying and suggested cram school re-enrollment. To support autonomy, OTR advised parents to wait for the child to initiate participation. They were encouraged to expand the child's opportunities.
Phase 4	April to March, 3rd Year of Junior High	Showed reduced interpersonal tension and increased study motivation. Compared to the early phase of OT, he developed greater self-awareness and began accepting that "it’s okay to fail."	Goal: Develop career goals and enhance school attendance toward graduation. Plan: Proposed separate classroom attendance. During sessions, OTR asked about school experiences to stabilize emotions and supported mood fluctuations.		Gradually transitioned from separate to regular classroom attendance, focusing on preferred subjects. Despite occasional discouragement over assignments due to perfectionism, he learned to pace himself.	Recommended parent-teacher collaboration for reentry into junior high. Since the school was integrated, OTR advised discussing required credits for graduation and explained associated risks and strategies to prevent anxiety,
Phase 5	April–July, 1st Year of High School	Demonstrated early self-understanding and problem-solving skills. No longer became emotionally distressed by failure; anxiety symptoms resolved.	Goal: Support high school graduation and manage academic stress. Plan: Due to post-advancement difficulties, OTR proposed transferring to a correspondence school. Support focused on maintaining motivation and helping with the transfer process.		Eventually consulted the OTR about academic struggles and expressed emotional instability. He and his parents decided to forgo internal high school graduation and transferred to a correspondence school, where he adjusted well.	OTR encouraged the family to visit correspondence high schools and complete enrollment procedures.
Completed visiting OT; July, 1st Year of High School						

The child's reversed sleep-wake cycle improved within two months, and he began attending to grooming during visits. Four months in, a paper labeled “Occupational Therapist” appeared on his wall, and he asked, “How can I become an OTR?” This suggested that the OTR’s presence influenced his future aspirations. Career conversations expanded to high school plans.

He expressed a desire to remain in his current junior high school. Despite motivation fluctuations, he gradually resumed self-study. The OTR suggested, “How about trying a cram school for your favorite subject, just once a week?” Ten months in, the child began attending cram school twice weekly, enabling broader social participation.

By April of his third year (11 months after the start), he began attending short school sessions in a separate room two times per week. However, anxiety increased due to underwhelming test results. The OTR supported him with discussions on mindset and self-acceptance.

This approach respected his wishes and encouraged self-determination. He chose to advance internally, as hoped. By month 22, he began attending high school daily, but struggled with class pace and workload. He told the OTR, “I want to transfer high schools.” The OTR introduced several correspondence schools, encouraging visits. The child appeared motivated and not distressed. In June, 25 months into the intervention, he transferred to a correspondence high school.

He later graduated from both high school and university and fulfilled his dream of becoming an OTR. Vineland-II scores in the Socialization Domain improved, and Maladaptive Behavior scores declined (Table 2).

**Table 2:** Vineland II adaptive behavior scores (Case 1) Vineland-II V-scores (mean = 15, SD = 3) indicate adaptive functioning in each behavioral subdomain. Increases in scores reflect improvements in the corresponding areas of daily living or social behavior.

Domain	Start of visiting the occupational therapist	Junior High graduation
Interpersonal Relationships	9	17
Play and Leisure Time	11	17
Coping Skills	15	16
Internalization	24	14
Externalization	16	14

Case study 2

A 13-year-old male child living with his mother and two older sisters was diagnosed with ADHD and ASD, and was using the authors’ day support service since his first year of elementary school. In July of his first junior high school year, home-visit OT by an OTR was initiated at his primary physician’s request, as the child had "encountered many difficulties at school; support for interpersonal skills and social competence is needed.”

Because he had previously used a day support service, he already had an established relationship with the visiting OT from the outset. Initially, he was attending school, and the OT supported his independence in tasks like organizing assignments, tidying his room, and fostering mental stability. Assignment management and schedule adjustment were also key intervention areas.

During his first year, the child maintained positive peer relationships and attended school regularly. However, after a class reshuffle in his second year, school attendance declined. He attributed this to teasing by classmates. Before summer vacation, he attended about three days a week but became fully absent afterward.

Table 3 outlines the characteristics of the child, the OT support process, and family relationships. OT visits increased from once to twice weekly, with efforts to visit in the morning, but the child was often asleep and unresponsive. He resisted school-related topics and often blamed others for his absences. Attempts to accompany him to school triggered physical symptoms such as stomachaches. To reset his routine, he was hospitalized from December of his second year through the winter break (four months after visits began).

**Table 3:** Intervention process for the child and his mother (Case 2) OTR: Occupational Therapists Registered

Phase	Period	Key Characteristics and Challenges in Case 2	OTR Intervention Goals and Plan	OTR Intervention and Patient Response	Support for and Relationship with Family (Mother)
Phase 1	September–December, 2nd Year of Junior High	Displayed high impulsivity and attention-seeking behaviors; frequently stole or misused family money. Daily life was centered around gaming and isolation in his room, disrupting his routine. Experienced psychosomatic symptoms (e.g., stomachaches) when attempting school attendance.	Goal: Improve daily rhythm and become a reassuring presence. Plan: OTR conducted twice-weekly morning visits. Built rapport through game discussions and empathized with his experiences of being bullied to build trust and alliance.	Initially became withdrawn when school was discussed, often blaming teachers or peers. Despite reminders about morning routines and school readiness, little improvement occurred early on.	The child's mother, an OT herself, did not exhibit excessive anxiety over his nonattendance. She and the OTR stayed in regular contact with school teachers, sharing updates and strategies.
Phase 2	January, 2nd Year of Junior High – July, 3rd Year of Junior High	Spent OT visits playing games while remaining bedridden; avoided cleaning his room. Used stolen money for in-game purchases. Formed lazy habits and distorted views, perceiving all teachers and peers as adversaries and believing he had no allies	Goal: Promote tidiness and challenge cognitive distortions. Plan: Focused on cleaning his room to maintain basic living standards. Worked on reshaping his views on communication and emphasized the value and difficulty of earning money	Though occasionally falling asleep during sessions, he never refused visits. He voiced negative views of school staff, resisting efforts to correct his perceptions.	Concerned about his living habits, the mother sometimes dismissed his emotional expressions. Due to lack of evidence, the family avoided direct confrontation over money theft and instead created a cash-free home environment. OTR advised her not to over-criticize and emphasized keeping a balanced emotional distance.
Phase 3	August–November, 3rd Year of Junior High	Lacked future goals and struggled with long-term thinking. Became mentally stable once summer vacation started, as the pressure to attend school was lifted	Goal: Develop a vision for high school and understand societal structure Plan: To shift his outlook on entrance exams, the OTR helped research high school systems and engaged in reflective discussions about his current status and future in society.	Gradually became receptive to discussions about high school and sought exam advice on his own. Possibly due to boredom, he stopped making in-game purchases.	OTR updated the mother on the childs progress and shared high school entrance information. They agreed to wait for the child to independently decide his preferred school.
Phase 4	December– March 3rd Year of Junior High	Showed reduced externalization and began recognizing personal desires and setting goals. Gradually resumed school attendance with the aim of progressing to high school	Goal: Promote self-initiated actions toward high school advancement. Plan: Encourage the child to attend junior high school as consistently as possible. Offered emotional support to help shift his mindset without placing blame for missed days.	Attendance improved through accommodations such as separate classroom use. He initiated visits to prospective high schools and engaged in club activities. He also voiced fewer complaints about peers and teachers, reducing unnecessary conflict.	OTR and the mother discussed how best to support the child’s transition to high school. The OTR emphasized the importance of self-determination and advised allowing the child to make the final decision independently.

Following discharge, he briefly resumed school attendance. However, by March of that year (seven months in), his daily rhythm deteriorated again, and he remained in bed much of the day. His room was unclean, with scattered dishes and trash, prompting the OT to assist with cleaning and maintaining basic living standards. The OT respected his interests and regularly checked on his living conditions. Still, he was hospitalized again in July of his third year (11 months in).

After discharge, the child was mentally stable during summer break, and the OT began career guidance. Initially, he showed little motivation, saying, “It’s fine if I’m a NEET (Not in Education, Employment, or Training).” The OT continuously discussed the importance of social engagement and future options. As a result, in September (13 months in), he began attending school two-three times per week.

Around this time, the child developed an interest in marine sports. The OT suggested visiting a high school with a canoeing club. After the visit, he set a goal: “I want to compete nationally.” He chose to apply to a public high school with a canoeing program instead of a correspondence school. He maintained attendance three to four days weekly and was accepted into his preferred school.

In high school, the child joined the canoeing club and continued attending without absences. In his second and third years, he won the prefectural championship and competed nationally.

Vineland-II socialization scores improved, as did scores in the Maladaptive Behavior Domain (Table 4).

**Table 4:** Vineland II adaptive behavior scale scores (Case 2) Vineland-II V-scores (mean = 15, SD = 3) indicate adaptive functioning in each behavioral subdomain. Increases in scores reflect improvements in the corresponding areas of daily living or social behavior.

Domain	Start of visiting the occupational therapist	Junior High graduation
Interpersonal Relationships	8	11
Play and Leisure Time	7	13
Coping Skills	5	13
Internalization	21	14
Externalization	24	18

## Discussion

The two cases shared several core features but also exhibited distinct differences that shaped the direction of support.

Common points and initial effects of home-visit OT

A key commonality was severe social isolation and disrupted daily rhythms due to school maladaptation. At the start of the intervention, both students had reversed day-night cycles, impaired self-care, and a strong tendency to avoid interaction. Home-visit OT began by building trust and restructuring daily routines. The OTR’s direct engagement in the clients’ home environments, reintroducing daytime activities and organizing spaces, reflects a unique advantage of home-based support. Over time, both students began setting goals independently, aided by emotional expression and enhanced self-understanding, which expanded their social participation. Trust built through the OTR relationship and engagement in non-school activities, such as cram school and club participation, could have facilitated improvements in social competence and coping. Corresponding improvements in Vineland-II scores across interpersonal, leisure, and coping domains further demonstrate OT’s role in enhancing social functioning in daily life.

Individualized support focus based on specific needs

Differences in Vineland-II score patterns revealed the need for tailored approaches based on each student’s developmental and psychological profile (Table 5). In Case 1, the child already had relatively high baseline scores (e.g., Interpersonal Relationships 9→17, Leisure Activities 11→17), indicating pre-existing social skills and introspective ability. His coping skills remained high (15→16), while support promoted self-awareness and future aspirations. Marked improvement in internalizing and externalizing behaviors (24→14 and 16→14) indicated significant gains in emotional regulation and interpersonal adaptability.

**Table 5:** Comparison of Vineland adaptive behavior scales II scores Higher Vineland-II V-scores indicate stronger adaptive functioning, and decreases in maladaptive behavior scores reflect improvement.

Domain	Case 1 (Pre→Post)	Case 2 (Pre→Post)	Interpretation
Interpersonal Relationships	9→17	8→11	Case 1 high baseline, Case 2 moderate improvement
Play and Leisure Time	11→17	7→13	Both improved; Case 2 large gains
Coping Skills	15→16	5→13	Case 1 stable high; Case 2 major increase
Internalizing	24→14	21→14	Both improved
Externalizing	16→14	24→18	Case 2 continues high dysregulation

Vineland-II V-scores are standardized with a mean of 15 and a standard deviation of 3, and changes of approximately 3 points are often regarded as clinically meaningful in group-based research. However, in students with ER-SAC, even smaller score increases, such as 1-point changes, could reflect meaningful improvements in daily adaptive functioning, given the sensitivity of V-scores to real-life behavioral changes.

In contrast, in Case 2, the child's initial scores were lower (Interpersonal Relationships 8→11, Leisure Activities 7→13, Coping Skills 5→13), and he struggled with impulsivity, distrust, and externalizing behaviors early on. A defining aspect of his progress was the considerable improvement in social skills after establishing a stable relationship with the OT and setting self-directed goals (e.g., aiming for national canoeing competitions). However, his maladaptive behavior scores, especially for externalizing issues, remained relatively high (24→18), suggesting ongoing challenges in emotional regulation. While the child in Case 1 supported emphasized introspection and professional development, the child in Case 2 focused on reshaping his perception of others and clarifying goals around high school enrollment. Both progressed toward social engagement and goal-directed behavior, though the structure and priorities of support differed significantly.

Phased support development and consistency with international frameworks

In both cases, support followed a phased process: from "isolation and withdrawal" to trust-building with the OTR, followed by "emotional expression" and "daily life restructuring," and eventually to "active social engagement" and "future planning." This trajectory aligns with international models for supporting socially withdrawn youth and mirrors the CHIME framework [15], which outlines mental health recovery components [17-20]. Specifically, elements such as "Connectedness," "Hope and optimism about the future," "Identity," "Meaning in life," and "Empowerment," were reflected throughout the intervention process, highlighting the relevance and efficacy of sustained, adaptable home-visit OT.

Future perspectives

The two cases presented in this study highlight the strong potential of home-visit OT as an effective intervention for students with ER-SAC. Support must be structured around each student’s unique psychosocial profile; standardized intervention stages are not suitable. Home-visit OT enables flexible, ongoing support grounded in daily life and holds clinical value as a means of promoting broad social reintegration, beyond simply facilitating school reentry.

## Conclusions

This study explored the progress and outcomes of home-visit OT for two junior high school students experiencing ER-SAC. In both cases, intervention began by establishing trust and restoring daily routines, followed by emotional expression, increased self-awareness, and eventual re-engagement in social life. These findings support the use of home-based OT not only for aiding school return but also for fostering broader social reintegration and role development.

While both students showed improvement, the progression and focus of support varied based on individual psychological traits and behavioral responses. This suggests that OTs can promote long-term recovery and social inclusion by recognizing when clients are psychologically ready to receive support and initiate change, then gradually adjusting goals accordingly. The support model described offers practical insight for working with students facing similar challenges. It emphasizes the necessity of adaptable support plans tailored to emotional, cognitive, and social needs rather than relying on uniform approaches. These findings could be viewed as illustrative rather than generalizable, as the two students with ER-SAC presented diverse emotional and psychosocial backgrounds.
